# Prognostic significance of *KRAS*, *NRAS*, *BRAF*, and *PIK3CA* mutations in stage II/III colorectal cancer: A retrospective study and meta-analysis

**DOI:** 10.1371/journal.pone.0320783

**Published:** 2025-04-25

**Authors:** Di Kang, Jing Li, Yangyang Li, Jingquan Xu, Jianlei Yang, Zili Zhang

**Affiliations:** Department of General Surgery, Tianjin Third Central Hospital, The Third Central Clinical College of Tianjin Medical University, Tianjin Key Laboratory of Extracorporeal Life Support for Critical Diseases, Artificial Cell Engineering Technology Research Center, Tianjin Institute of Hepatobiliary Disease, Tianjin, China; Osaka International Cancer Institute: Osaka Kokusai Gan Center, JAPAN

## Abstract

The prognostic significance of *KRAS* and *BRAF* mutations is well-established in metastatic colorectal cancer (CRC) but remains uncertain in early-stage tumors. This study retrospectively analyzed 47 stage II/III CRC patients undergoing curative surgery to assess the association of mutations in *KRAS*, *NRAS*, *BRAF*, and *PIK3CA* with overall survival (OS) and disease-free survival (DFS). Additionally, a meta-analysis was conducted to validate the prognostic relevance of these gene mutations. We included post hoc analyses of phase III randomized controlled trials (RCTs) in stage II/III patients receiving adjuvant therapy after curative resection in the meta-analysis. Pooled hazard ratio (HR) and 95% confidence interval (CI) was calculated using a random-effect model in the overall population, stratified subgroups adjusted for microsatellite instability (MSI) status, and within MSI-high (MSI-H) and microsatellite-stable (MSS) populations. In the retrospective cohort, mutations in *KRAS*, *NRAS*, *BRAF*, and *PIK3CA* were identified in 29.8%, 4.3%, 8.5%, and 14.9% of patients, respectively. No significant association between individual genes and survival was observed. However, in MSS patients, concurrent mutations were significantly associated with shorter OS and DFS (log-rank test, P < 0.05). The meta-analysis incorporated 13 eligible studies, including 15,034 patients. Pooled analyses revealed that *KRAS* and *BRAF* mutations were significantly linked to poor OS (*KRAS*: HR = 1.25, 95%CI: 1.06-1.47, P = 0.008; *BRAF*: HR = 1.43, 95%CI: 1.26-1.63, P < 0.001) and DFS (*KRAS*: HR = 1.36, 95%CI: 1.21-1.53, P < 0.001; *BRAF*: HR = 1.21, 95%CI: 1.02-1.44, P = 0.032). The prognostic impact of *BRAF* mutation increased with MSI adjustment compared those without MSI adjustment. In MSS tumors, *KRAS*-mutant patients demonstrated significantly shorter DFS (HR = 1.63, 95%CI: 1.25-2.13, P < 0.001), while *BRAF*-mutant patients exhibited reduced OS (HR = 1.53, 95%CI: 1.24-1.89, P < 0.001) and DFS (HR = 1.72, 95%CI: 1.20-2.46, P = 0.003) compared to wildtype patients. Conversely, no significant survival differences were found between mutant and wildtype patients in the MSI-H population. Although *PIK3CA* mutation was nominally associated with OS (HR = 0.86, 95%CI: 0.75-1.00, P = 0.046), the pooled result lacked robustness. In conclusion, *KRAS* and *BRAF* mutations had a negative prognostic impact on MSS stage II/III CRC patients receiving adjuvant therapy following curative resection. These patients may benefit from more effective adjuvant treatment strategies.

## Introduction

Colorectal cancer (CRC) is the most common malignancy of the digestive tract, ranking as the third most prevalent cancer worldwide and the second leading cause of cancer-related mortality [1]. In China, both the incidence and mortality rates of CRC have been steadily increasing [2]. In 2010, there were 274800 newly diagnosed CRC cases and 132100 CRC-related deaths [3]. It is estimated that by 2025, these numbers will rise 642300 new cases and 221100 deaths [4].

Tumor staging remains the most crucial prognostic factor for CRC. For patients with stage III or high-risk stage II CRC, adjuvant chemotherapy following curative resection is the standard protocol that significantly reduces the risk of recurrence and mortality [5–7]. However, even among patients with the same tumor stage, prognosis can vary widely [8]. The identification of various genetic alterations in CRC highlights the importance of establishing reliable genetic prognostic markers to guide treatment strategies and improve both survival rates and quality of life [9, 10].

Aberrant activation of the RAS-RAF-MAPK and PI3K-PTEN-AKT pathways promotes tumor cell proliferation, invasion, metastasis, and angiogenesis. Mutations in pathway-related genes, such as *KRAS*, *NRAS*, *BRAF*, and *PIK3CA*, influence treatment responses and serve as key prognostic indicators in cancer treatment [11, 12]. In metastatic colorectal cancer (mCRC), *KRAS* and *BRAF* mutations are well-known markers of resistance and poor prognosis in patients receiving anti-epidermal growth factor receptor (EGFR) monoclonal antibodies [13–16]. However, the prognostic value of these mutations in patients with stage II/III CRC undergoing curative surgery followed by adjuvant chemotherapy remains controversial. For instance, a post hoc analysis of the phase III CALGB 89803 trial by Ogino *et al*. found no significant association between *KRAS* mutation and overall survival (OS) or disease-free survival (DFS), although *BRAF* mutation was linked to shorter OS [17]. Analyses of the NSABP C-07 and C-08 phase III trials by Gavin *et al*. produced similar results [18]. In contrast, post hoc analyses of the NCCTG N0147 and PETACC-8 trials indicated that *KRAS* mutation was significantly associated with worse OS and DFS [19,20].

In this retrospective study, we evaluated the prognostic value of *KRAS*, *NRAS*, *BRAF*, and *PIK3CA* mutations in Chinese patients with stage II/III CRC. Additionally, we conducted a systematic review and meta-analysis to assess the prognostic significance of these gene mutations in stage II/III CRC patients who received adjuvant chemotherapy following curative surgery.

## Materials and methods

### Participants

This retrospective study included CRC patients who underwent curative resection surgery at Tianjin Third Central Hospital, Tianjin, China between December 2019 and March 2022. Patients were eligible for analysis if they had a confirmed diagnosis of stage II and III CRC according to the 8th Edition of the American Joint Committee on Cancer (AJCC) and complete clinical, pathological, and survival data. Exclusion criteria included: (1) prior chemotherapy, radiotherapy, or targeted therapy before surgery; (2) familial adenomatous polyposis or Lynch syndrome; and (3) colorectal tumor metastasized from tumors in other organs. The date when data were accessed for research purposes was 10/7/2024. All researchers had no access to information that could identify individual participants during or after data collection. A total of 47 stage II/III CRC patients were included in this study. The study was conducted in accordance with the Declaration of Helsinki and approved by the Ethics Committee of Tianjin Third Central Hospital. Written informed consent was obtained from all patients. The raw data of this retrospective analysis were supplied in S1 Data.

### Analysis of KRAS, NRAS, BRAF, PIK3CA, and MSI status

Mutation status of *KRAS, NRAS, BRAF*, and *PIK3CA* genes was determined from genomic DNA extracted from formalin-fixed, paraffin-embedded (FFPE) samples using the QIAamp DNA FFPE Tissue Kit (Qiagen, Germany). Targeted next-generation sequencing (NGS) was performed using a pan-cancer panel covering all exons and critical introns of 616 cancer-related genes. DNA (50-100ng) was used for library construction with the MGIEasy Universal DNA Library Kit (MGI, China), followed by hybrid capture using an xGen Hybridization and Wash Kit (IDT, USA). Libraries were sequenced with 2 × 100 bp paired-end reads on the MGISEQ-2000 (MGI, China) platform. Sequencing data were processed to detect single nucleotide variations (SNVs) and short insertions and deletions (indels). Reads were aligned to the human reference genome GRCh37/hg19 using BWA-MEM v0.7.17. VarScan v 2.4.3 was used to call SNVs and indels, requiring at least 5 supporting reads for indels and 8 supporting reads for SNVs.

Microsatellite instability (MSI) status was assessed using six monomorphic mononucleotide markers (NR-21, BAT-26, NR-27, BAT-25, NR-24, MONO-27). Tumors were classified as MSI-high (MSI-H) if two or more markers were unstable, and microsatellite stable (MSS) if fewer than two markers were unstable.

### Literature search and selection process for meta-analysis

To evaluate the prognostic value of *KRAS*, *NRAS*, *BRAF*, and *PIK3CA* mutations in stage II and III CRC patients receiving adjuvant therapy post-surgery, a preferred reporting items for systematic reviews and meta-analyses (PRISMA)-compliant meta-analysis was conducted (S1 Checklist) [21]. Only post hoc analyses from phase III randomized controlled trials (RCTs) were included, while observational studies were excluded to minimize biases related to mutation detection, patient selection, adjuvant therapy, and selective reporting. A comprehensive literature search was conducted using PubMed, EMBASE, Web of Science, and the Cochrane Library, covering studies published until June 30, 2024. Search strategies for these databases are detailed in S1 Table. Only articles in English were considered, and reference lists of relevant studies were manually reviewed for additional eligible publications.

**Fig 1:**
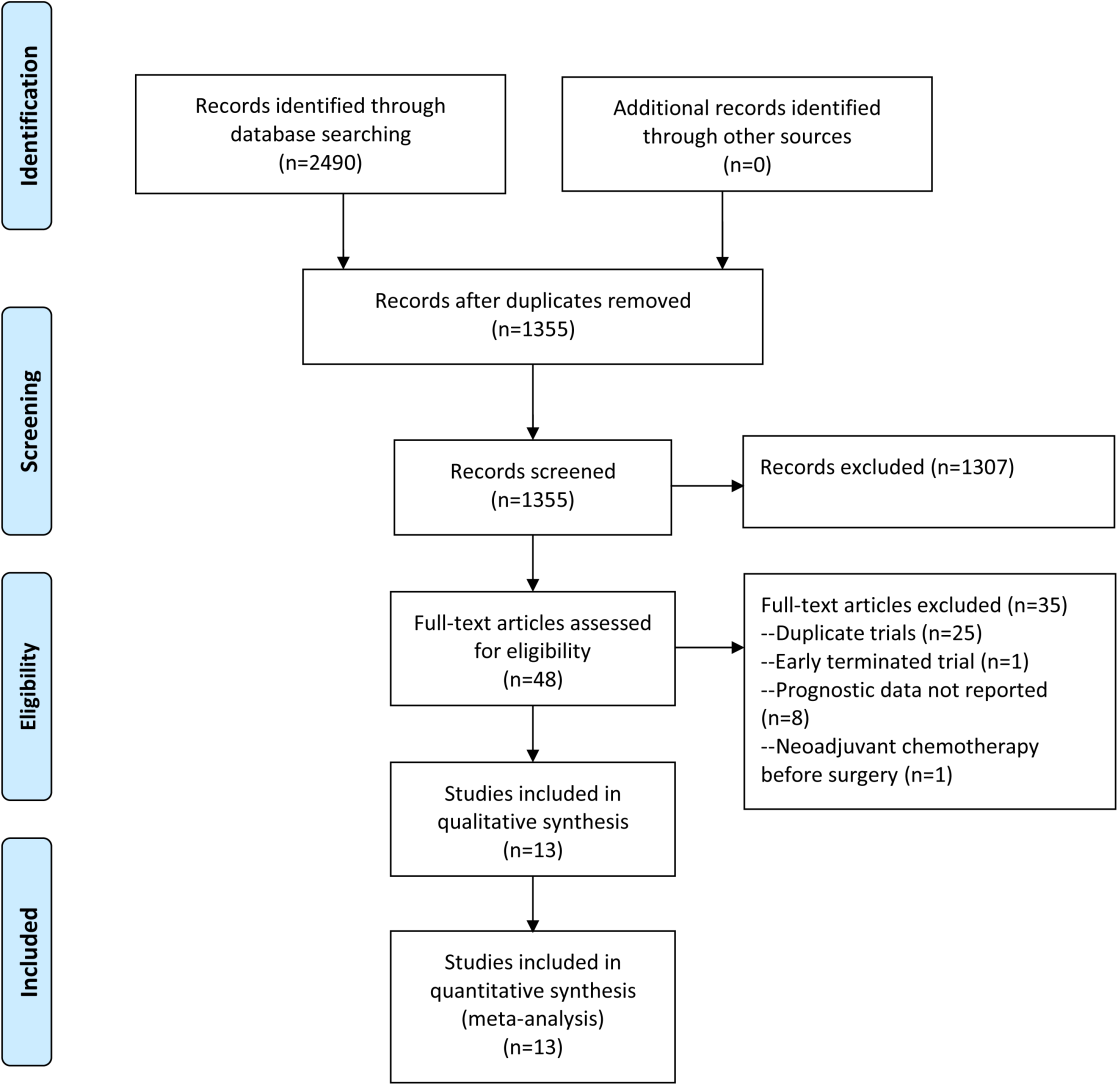
Flowchart of literature search and selection for meta-analysis.

The eligibility of studies retrieved from literature search was assessed using the PICOS framework: Participants (P) were stage II and III CRC patients receiving adjuvant therapy post-resection; Intervention (I) was the presence of *KRAS*, *NRAS*, *BRAF*, or *PIK3CA* mutations; Control (C) was wild-type status for these genes; Outcomes (O) were OS and DFS; Study design (S) was post-hoc analysis of phase III RCTs. Observational studies, reviews, case series, studies of advanced tumors or mixed stages (I–IV), and those lacking sufficient prognostic data were excluded. For multiple post-hoc analyses from the same trial, only the analysis with the most complete data was included. The process of literature search and selection is shown in Fig 1.

### Quality assessment and data extraction

Study quality was assessed using the Newcastle-Ottawa Scale (NOS) for cohort studies [22]. A maximum of 9 stars was awarded based on selection, comparability, and outcome categories. Studies with 7 or more stars were considered high quality, those with 5 or 6 stars moderate quality, and those with 4 or fewer stars low quality.

The following information was extracted: first author, publication year, trial name, adjuvant therapy, cancer type, tumor stage, number of patients analyzed, number of *KRAS, NRAS, BRAF* or *PIK3CA*-mutant patients, follow-up duration, and hazard ratios (HRs) with 95% confidence interval (CIs) for OS and DFS. OS was defined as the time from randomization to death from any cause or the last follow-up. DFS was defined as the time from assignment to cancer recurrence, the occurrence of a new colorectal tumor, death from any cause, or last follow-up. When available, HRs from multivariate analysis adjusted for MSI status were prioritized; otherwise, HRs from univariate analysis were used. Relapse-free survival (RFS) was considered equivalent to DFS.

Two independent authors (DK, JL) conducted the literature search and selection, data extraction, and quality assessment. Discrepancies were resolved through discussion with a third author (JY).

### Statistical analysis

In the retrospective analysis, categorical variables were compared using the chi-square test or Fisher’s exact test. Kaplan-Meier (KM) survival curves were generated, and survival differences were evaluated using the log-rank test. In addition to single gene mutation, the impact of concurrent mutations defined as simultaneous mutations in at least two of these four genes on survival was analyzed. Univariate and multivariate Cox regression analyses were conducted to calculate HR and 95%CI. For the meta-analysis, between-study heterogeneity was assessed using the I^2^ statistic. A random-effects model was applied for pooled analyses, regardless of heterogeneity, to generate more conservative estimates than a fixed-effects model. Pooled HRs with 95%CIs were calculated to assess the prognostic impact of *KRAS, NRAS, BRAF* and *PIK3CA* mutations on survival outcomes. Subgroups analyses were conducted based on whether HR estimates were adjusted for MSI status. Studies performing multivariate analysis with adjustment of MSI status were included in the subgroup with MSI adjustment. Studies only conducting univariate analysis or performing multivariate analysis without adjustment of MSI status were included in the subgroup without MSI adjustment. Further analyses were performed separately in MSI-H and MSS patients. Sensitivity analyses, using a leave-one-out mothed, were conducted by omitting one study at a time to assess the robustness of the results. Funnel plots and Egger’s tests were applied to detect potential publication bias. All statistical analyses were conducted using STATA 16.0 (StataCorp, US). P value < 0.05 was considered statistically significant.

## Results

### Prognostic value of KRAS, NRAS, BRAF and PIK3CA mutations in retrospective analysis

In the cohort of 47 CRC patients, 29 (61.7%) were male, and 20 (38.3%) were female, with ages ranging from 37 to 84 years and a mean age of 62.9 years. Twenty-six (55.3%) patients had stage II tumor, and 21 (44.7%) had stage III tumor. The resection margins of all patients were microscopically clear of malignant cells (R0 resection). The median number of harvested lymph nodes was 19 (interquartile range: 12-26). As shown in Table 1, the mutation rates of *KRAS*, *NRAS*, *BRAF*, and *PIK3CA* were 29.8%, 4.3%, 8.5%, and 14.9%, respectively. Four patients had concurrent *KRAS* and *PIK3CA* mutations, and one had concurrent *BRAF* and *PIK3CA* mutations. The mutation distribution is shown in S2 Table. Regarding MSI status, there were 4 MSI-H and 43 MSS patients. The clinicopathological features were similar between *KRAS* mutant and wildtype patients except for tumor location (Table 1). *KRAS* mutations were more frequently detected in the right-sided tumor than left-sided tumor (P = 0.026).

**Table 1:** Association between *KRAS* mutation and clinicopathological features in CRC patients.

Clinicopathological features	Total (n = 47)	*KRAS*, n (%)		P
		Mutant (n = 14)		Wildtype (n = 33)
Sex				0.282
Male	29	7 (24.1)	22 (75.9)	
Female	18	7 (38.9)	11 (61.1)	
Age, years				0.235
>60	31	11 (35.5)	20 (64.5)	
≤60	16	3 (18.8)	13 (81.2)	
Location				0.026
Left side	34	7 (20.6)	27 (79.4)	
Right side	13	7 (53.3)	6 (46.2)	
Differentiation				0.336
Well to moderate	36	12 (33.3)	24 (66.7)	
Poor to undifferentiation	11	2 (18.2)	9 (81.8)	
TNM stage				0.870
II	26	8 (30.8)	18 (69.2)	
III	21	6 (28.6)	15 (71.4)	
Number of harvested lymph nodes, median (IQR)	19 (12-26)	25 (14-30)	18 (12-24)	0.289
*KRAS* mutant	14	–	–	
*NRAS* mutant	2	0 (0)	2 (100)	>0.999 ^#^
*BRAF* mutant	4	0 (0)	4 (100)	0.302 ^#^
*PIK3CA* mutant	7	4 (57.1)	3 (42.9)	0.086
MSI-H	4	1 (25.0)	3 (75.0)	>0.999 ^#^

# Fisher’s exact test.

CRC: colorectal cancer; IQR: interquartile range; MSI-H: microsatellite instability-high.

**Fig 2:**
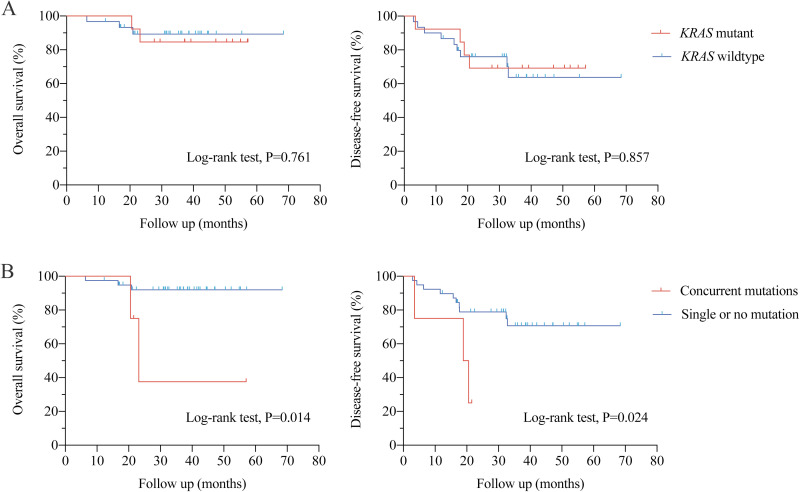
Kaplan-Meier plot for survival analysis in MSS population. (A) **KRAS mutation;** (B) **Concurrent mutations in KRAS, NRAS, BRAF, and PIK3CA.**

The median follow-up was 35.3 months (range: 6.4-68.4 months). Cox regression analysis demonstrated that tumor stage was an independent factor for DFS (S3 Table). No significant correlation between gene mutations and survival outcomes was observed. We performed additional survival analyses in 43 MSS patients (S4 Table). *KRAS* mutations were not significantly associated with OS or DFS (Fig 2A). Univariate analysis showed that patients with concurrent mutations had significantly worse OS and DFS compared to those with single or no mutations (log-rank test P < 0.05, Fig 2B).

### Baseline characteristics of studies included in meta-analysis

A total of 2490 records were identified through the literature search (Fig 1). After screening title, abstract, and full text, 13 eligible studies with a total of 15034 patients were included in the meta-analysis (S5 Table). These studies performed post-hoc analyses of phase III trials, including Intergroup 0135/NCCTG 91-46-53/NCIC CTG CO.9 [23], QUASAR [24], CALGB 89803 [17,25], NSABP C-07 and C-08 [18], PETACC-3 [26], ACTRN12610000509066 [27], NCCTG N0147 [19], MOSAIC [6], PETACC-8 [20], QUASAR2 [28], JCOG0910 [29], and CALGB/SWOG 80702 [30]. *KRAS* mutation status was analyzed in 9 studies, involving 11,818 CRC patients, of whom 4183 had *KRAS* mutations. The prognostic significance of *BRAF* mutation status was assessed in 11 studies, comprising 13210 CRC cases and 1418 *BRAF*-mutant individuals. Five studies assessed the association between *PIK3CA* mutation status and survival outcomes, with 5095 CRC patients and 961 *PIK3CA*-mutant individuals [18,25,28–30]. Only three studies, with 3271 cancer patients and 89 *NRAS*-mutant individuals, analyzed the association between *NRAS* mutation status and survival [18,28,29]. Seven trials adjusted for MSI status in their HR estimates [17,19,20,25,26,28,30]. Among these studies, one performed separate survival analysis in both MSI-H and MSS populations [20] and one study in MSS population [28]. Among the other studies that did not adjust for MSI status, three performed separate survival analyses in both MSI-H and MSS populations [20,23,24]. The baseline characteristics of included studies were summarized in Table 2. According to NOS assessment, all studies were considered to have high quality (S6 Table). The extracted data for quantitative analysis are shown in S2 Data.

**Table 2:** Baseline characteristics of studies included in meta-analysis.

Study	Trial name	Cancer	Adjuvant therapy	Pts in study/ original trial (%)	Mutated patients				Median follow-up	Outcome	MSI adjustment
					KRAS	NRAS	BRAF	PIK3CA			
French, 2008	Intergroup 0135/NCCTG 91-46-53/NCIC CTG CO.9	Stage II-III CC	High dose levamisole + 5FU/LV vs low dose levamisole + 5FU/LV	490/878 (55.8)	NR	NR	77	NR	NR	DFS, OS	No
Hutchins, 2011	QUASAR	Stage II-III CRC	5FU/LV vs observation	1551/3239 (47.9)	536	NR	124	NR	NR	DFS	No
Ogino, 2012	CALGB 89803	Stage III CC	5FU/LV vs IFL	506/1264 (40.0)	176	NR	75	NR	7.6 years	DFS, OS	Yes
Gavin, 2012	NSABP C-07, C-08	Stage II-III CC	5FU/LV + oxaliplatin vs 5FU/LV; FOLFOX + bevacizumab vs FOLFOX	2226/5202 (42.8)	793	59	316	422	NR	DFS, OS	No
Roth, 2012	PETACC-3	Stage II-III CC	5FU/LV vs FOLFORI	1404/3278 (42.8)	476	NR	103	NR	69 months	DFS, OS	Yes
Ogino, 2013	CALGB 89803	Stage III CC	5FU/LV vs IFL	627/1264 (49.6)	NR	NR	NR	74	7.6 years	DFS, OS	Yes
Pentheroudakis, 2015	ACTRN12610000509066	Stage II-III CRC	FOLFOX vs XELOX	321/441 (72.8)	133	NR	15	NR	74.7 months	DFS, OS	No
Sinicrope, 2015	NCCTG N0147	Stage III CC	FOLFOX + cetuximab vs FOLFOX	2974/3018 (98.5)	1042	NR	346	NR	4.9 years	DFS, OS	Yes
Andre, 2015	MOSAIC	Stage II-III CC	LV5FU2 vs FOLFOX	902/2246 (40.2)	NR	NR	94	NR	9.46 years	OS	No
Taieb, 2016	PETACC-8	Stage III CC	FOLFOX + cetuximab vs FOLFOX	1791/2559 (70.0)	588	NR	148	NR	3.52 years	DFS, OS	Yes
Domingo, 2018	QUASAR2	Stage II-III CRC	Capecitabine + bevacizumab vs capecitabin	511/1952 (26.2)	206	8	79	101	4.92 years	DFS	Yes
Shida, 2023	JCOG0910	Stage III CRC	Capecitabine vs S-1	534/1564 (34.1)	233	22	41	105	23.7 months	DFS	No
Nowak, 2024	CALGB/SWOG 80702	Stage III CC	FOLFOX + celecoxib vs FOLFOX	1197/2524 (47.4)	NR	NR	NR	259	NR	DFS, OS	Yes

5FU/LV: bonus 5-fluorouracil +  leucovorin; CC: colon cancer; CRC: colorectal cancer; DFS: disease-free survival; FOLFIRI: 5-fluorouracil +  folinic acid +  irinotecan; FOLFOX: 5FU/LV +  oxaliplatin; IFL: irinotecan +  bolus 5-fluorouracil +  leucovorin; LV5FU2: the so-called de Gramont regimen; MSI: microsatellite instability; NR: not reported; OS: overall survival; Pts: patients; XELOX: capecitabine +  oxaliplatin.

### Prognostic value of KRAS mutations in meta-analysis

**Fig 3:**
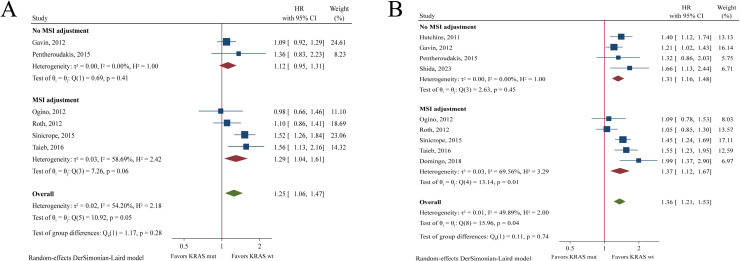
Forest plot of meta-analysis of the association between *KRAS* mutation and overall survival (A) and disease-free survival (B).

The pooled analysis showed that *KRAS* mutations were significantly associated with worse OS (HR = 1.25, 95%CI: 1.06-1.47, P = 0.008, Fig 3A) and DFS (HR = 1.36, 95%CI: 1.21-1.53, P < 0.001, Fig 3B). In the subgroup with MSI adjustment, the associations remained significant (OS: HR = 1.29, 95%CI: 1.04-1.61, P = 0.019; DFS: HR = 1.37, 95%CI: 1.12-1.67, P = 0.002). In the subgroup without MSI adjustment, *KRAS* mutation status was significantly associated with DFS (HR = 1.32, 95%CI: 1.16-1.48, P < 0.001) but not with OS (HR = 1.12, 95%CI: 0.95-1.31, P = 0.180).

**Fig 4:**
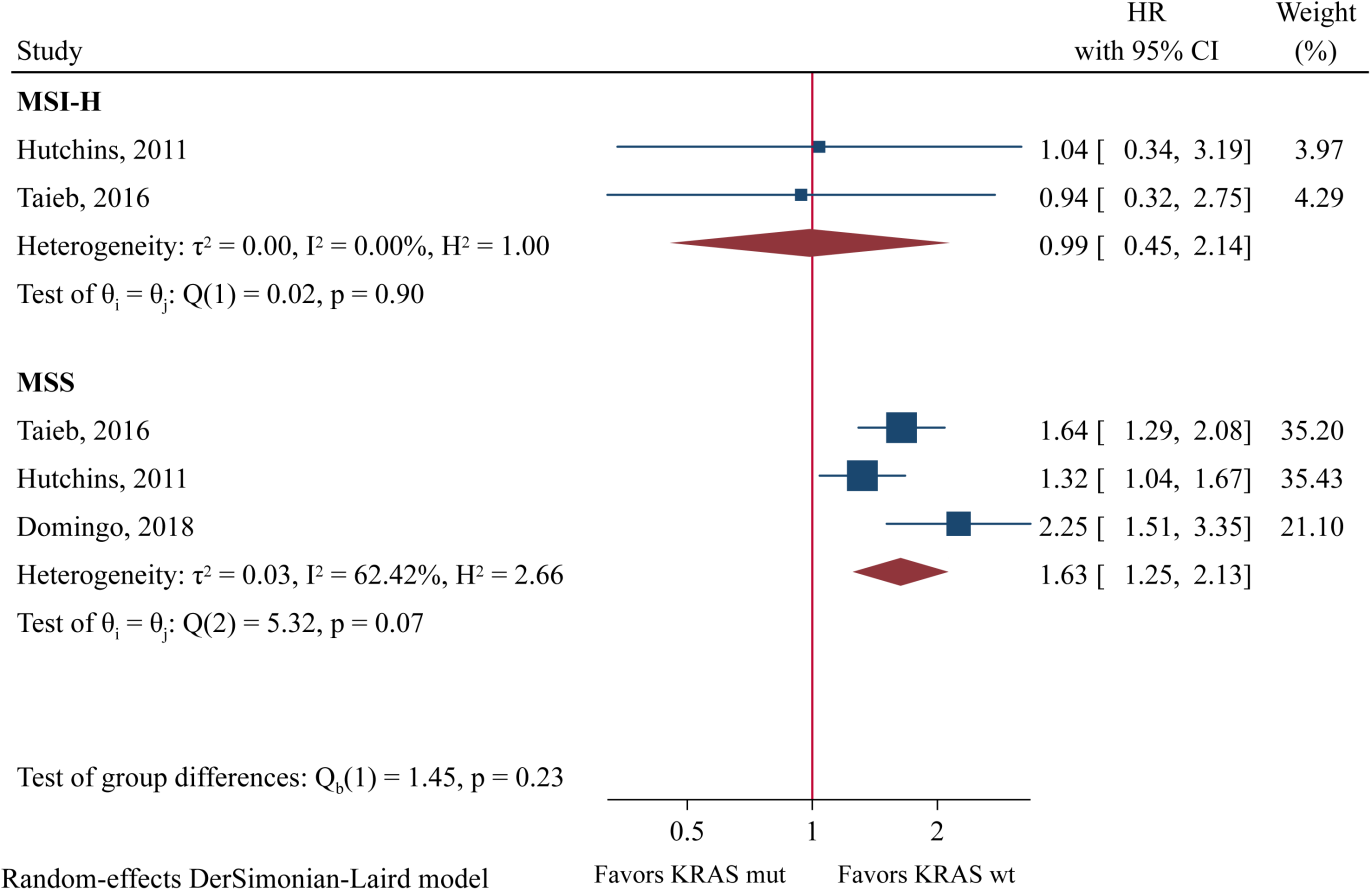
Forest plot of meta-analysis of the association between *KRAS* mutation and disease-free survival in MSI-H and MSS populations.

One study performed separate OS analyses in MSI-H and MSS populations [20], showing that *KRAS* mutation was significantly related with worse OS in MSS patients (HR = 1.71, 95%CI: 1.21-2.41) but not in MSI-H patients (HR = 0.90, 95%CI: 0.23-3.45). In terms of DFS (Fig 4), pooled analysis demonstrated that *KRAS* mutation status was a prognostic factor in the MSS population (HR = 1.63, 95%CI: 1.25-2.13, P < 0.001), but not in the MSI-H population (HR = 0.99, 95%CI: 0.45-2.14, P = 0.973).

### Prognostic value of BRAF mutations in meta-analysis

**Fig 5:**
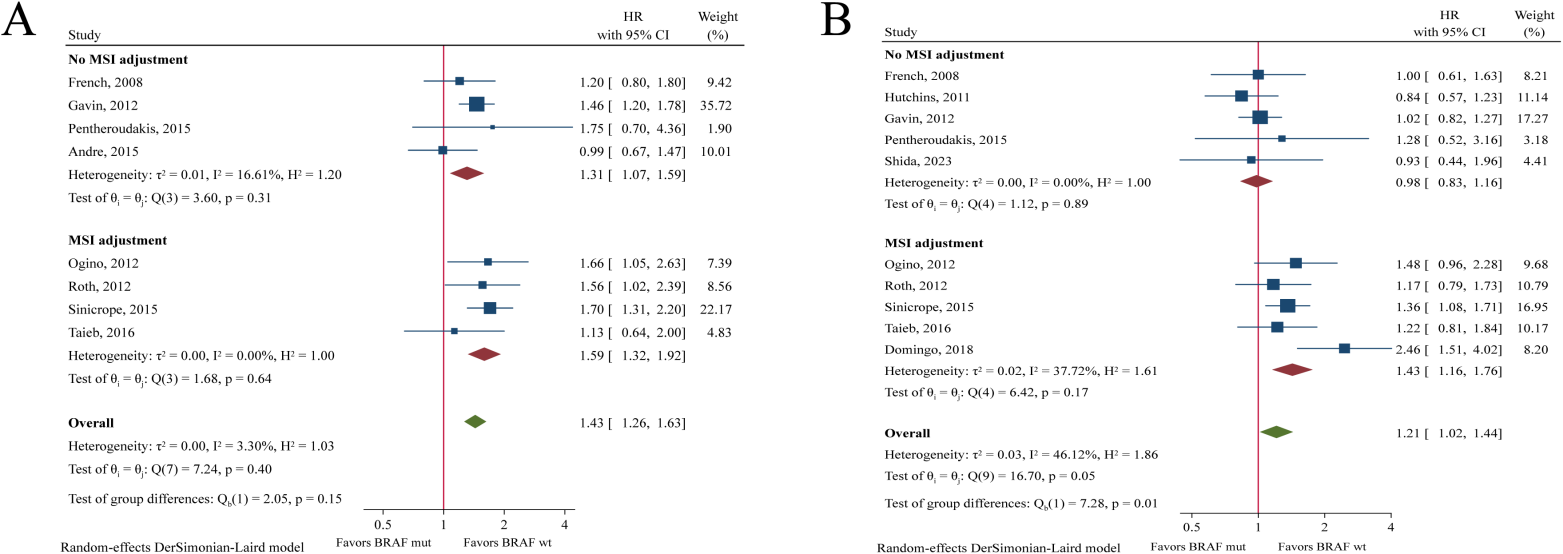
Forest plot of meta-analysis of the association between *BRAF* mutation and overall survival (A) and disease-free survival (B).

Meta-analysis revealed that *BRAF* mutations were significantly associated with worse OS (HR = 1.43, 95%CI: 1.26-1.63, P < 0.001, Fig 5A) and DFS (HR = 1.21, 95%CI: 1.02-1.44, P = 0.032, Fig 5B). In the subgroup with MSI adjustment, *BRAF*-mutant patients had significantly worse OS (HR = 1.59, 95%CI: 1.32-1.92, P < 0.001) and DFS (HR = 1.43, 95%CI: 1.16-1.76, P = 0.001) compared to wildtype patients. In the subgroup without MSI adjustment, *BRAF* mutations were significantly associated with OS (HR = 1.31, 95%CI: 1.07-1.59, P = 0.007) but not DFS (HR = 0.98, 95%CI: 0.83-1.16, P = 0.832).

**Fig 6:**
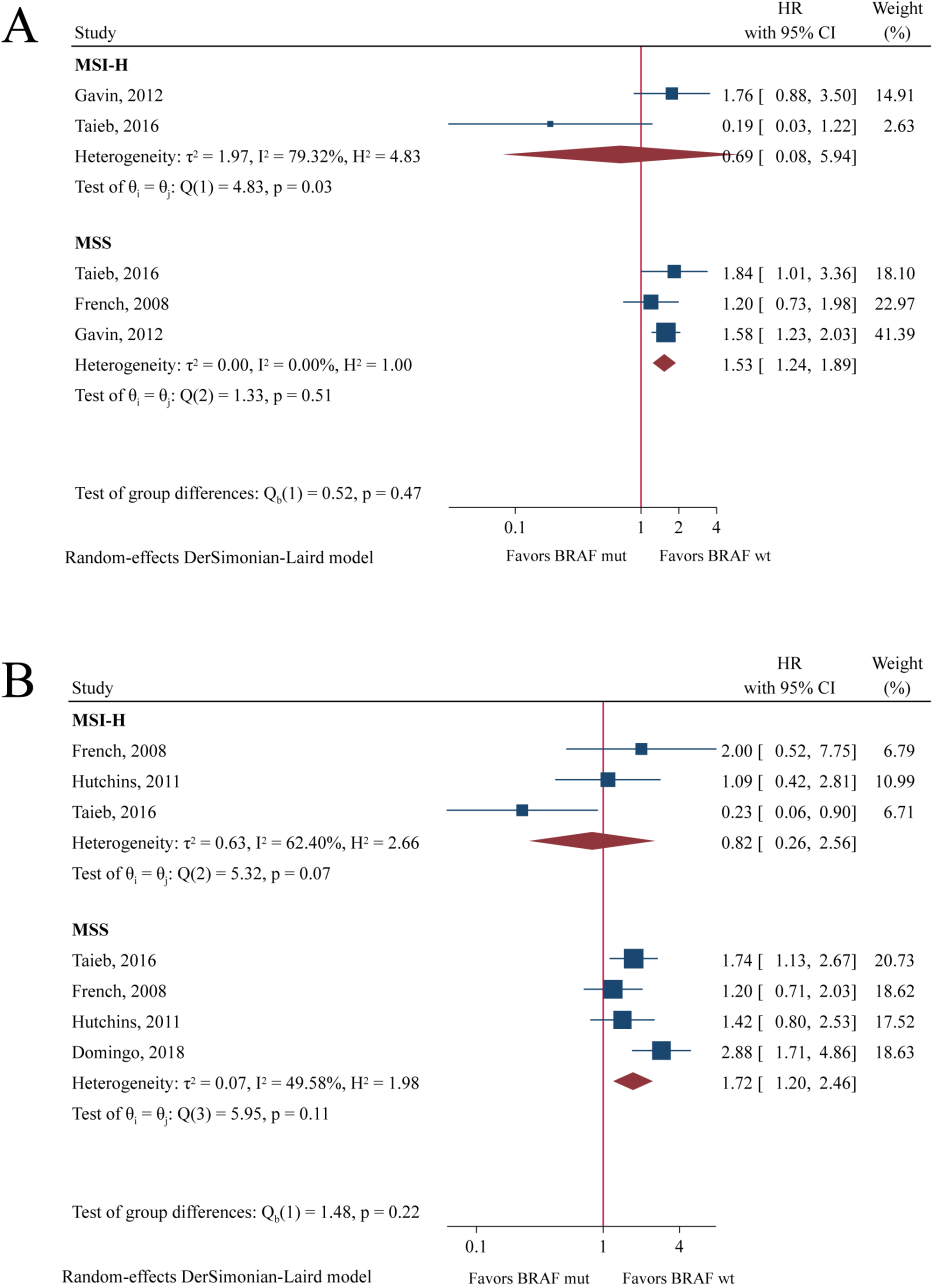
Forest plot of meta-analysis of the association between *BRAF* mutation and overall survival (A) and disease-free survival (B) in MSI-H and MSS populations.

As shown in Fig 6, *BRAF* mutation status was a prognostic factor in MSS population (OS: HR = 1.53, 95%CI: 1.24-1.89, P < 0.001; DFS: HR = 1.72, 95%CI: 1.20-2.46, P = 0.003), but not in MSI-H population (OS: HR = 0.69, 95%CI: 0.08-5.94, P = 0.734; DFS: HR = 0.82, 95%CI: 0.26-2.56, P = 0.731).

### Prognostic value of NRAS and PIK3CA mutations in meta-analysis

None of the trials evaluating *NRAS* mutations had adjusted for MSI status. *NRAS* mutations were not significantly associated with survival outcomes (S1 Fig). *PIK3CA* mutation was associated with favorable DFS at marginal significance (HR = 0.86, 95%CI: 0.75-1.00, P = 0.046, S2 Fig). However, no association between *PIK3CA* mutation and OS was found (HR = 0.91, 95%CI: 0.77-1.08, P = 0.266, S3 Fig).

### Sensitivity analysis and publication bias

Sensitivity analysis showed that omitting Domingo’s study reduced between-study heterogeneity of meta-analysis of *BRAF* mutation associated with DFS from 46.1% to 0% [28]. The association remained significant (HR = 1.14, 95%CI: 1.02-1.29, P = 0.027). In the analysis of *PIK3CA* on DFS, the association became insignificant (HR = 0.90, 95%CI: 0.76-1.05, P = 0.155) after excluding Nowak’s study [30]. No potential publication bias was observed in the analyses of *KRAS* and *BRAF* mutations (S4 Fig).

## Discussion

In the retrospective cohort, we did not observe a significant association between individual genes including *KRAS*, *BRAF*, *NRAS*, and *PIK3CA* and the survival outcomes of stage II/III CRC patients, possibly due to the small sample size. Notably, in patients with MSS tumors, concurrent mutations were associated with a poor prognosis. To confirm the prognostic value of these mutations, we conducted a meta-analysis by pooling post hoc analyses of phase III RCTs in stage II/III patients undergoing adjuvant therapy after surgery. The pooled analysis demonstrated that *KRAS* and *BRAF* mutations were significantly associated with worse OS and DFS. The prognostic significance was further modified by MSI status, with unfavorable prognoses observed only in MSS patients and not in those with MSI-H tumors.

The RAS-RAF-MAPK signaling pathway play a crucial role in regulating cell growth, differentiation, proliferation, and survival. Mutations in pathway-related genes, such as *KRAS*, *NRAS*, and *BRAF*, result in abnormal downstream signaling that drives uncontrolled cell growth, thereby contributing to tumorigenesis. The mutation frequency of *KRAS* in CRC is 35-45%, with the most common mutations occurring at codons 12 and 13 in exon 2, while fewer mutations are observed at codon 61 and codon 146 [31]. *NRAS* mutations are less frequent, occurring in 1-7% of cases [32,33]. *BRAF* mutations occur in 2-15% of CRC cases, with the V600E mutation accounting for over 90% of these mutations [34]. *PIK3CA*, another oncogene frequently mutated in CRC, contributes to cancer progression by activating the PI3K-AKT-mTOR pathway [35]. The mutation frequency of *PIK3CA* in CRC ranges from 10% to 30% [36,37]. In our study, the mutation rates of *KRAS*, *BRAF*, *NRAS*, and *PIK3CA* were 29.8%, 4.3%, 8.5%, and 14.9%, respectively, aligning with previous reports. Notably, *KRAS* and *BRAF* were mutually exclusive, whereas *PIK3CA* mutations frequently co-occurred with other genes, especially *KRAS* [38,39]. In our cohort, four out of seven *PIK3CA* mutations co-occurred with *KRAS* mutation and one with *NRAS* mutation.

The prognostic role of *KRAS* and *BRAF* mutations has been well-established in mCRC, where they guide patient selection for targeted therapies [40]. Patients with *KRAS* or *BRAF* mutations are typically excluded from treatment with cetuximab, an anti-EGFR antibody, due to inherent resistance [41]. However, the prognostic impact of these mutations in early-stage CRC has not been conclusively confirmed [42–44]. To derive more reliable conclusions, we performed a meta-analysis that pooled post hoc analyses from phase III trials, which typically offer higher quality evidence compared to retrospective cohort studies. Our analysis revealed that *KRAS* and *BRAF* mutations significantly increased the risk of tumor occurrence and death in stage II/III CRC patients. Additionally, we observed a strong interaction between *BRAF* mutations and MSI status, with a higher HR estimate for DFS in the MSI-adjusted subgroup compared to the non-MSI-adjusted subgroup (1.43 vs 0.98, P = 0.007), and a similar trend for OS (1.59 vs 1.31, P = 0.152). Separate analyses in MSS and MSI-H populations revealed that *KRAS* and *BRAF* mutations significantly worsened survival outcomes only in MSS patients (all P values < 0.01), while no significant association was found in the MSI-H population (all P values > 0.05). These results suggest that the negative prognostic impact of *KRAS*/*BRAF* mutations is confined to MSS tumors, whereas MSI-H status confers a more favorable prognosis regardless of mutation status. Therefore, assessing both MSI status and *KRAS*/*BRAF* mutation status can aid in more accurate prognosis management for stage II/III CRC patients.

Previous studies on the prognostic role of *PIK3CA* mutation in early stage CRC patients have produced inconsistent results [18,45]. Our meta-analysis suggested a marginally prolonged DFS in patients with *PIK3CA* mutation (HR = 0.86, 95%CI: 0.75-1.00, P = 0.046). However, this findings were not robust and appeared to be driven by Nowak’s study, where 23.4% of patients used low-dose aspirin [30]. Previous researches suggest that regular aspirin use after CRC diagnosis reduces the recurrence rate in *PIK3CA*-mutant patients but not in those without *PIK3CA* mutation [46]. When Nowak’s study was excluded, the association between *PIK3CA* mutation and DFS became non-significant (P = 0.155). *NRAS* mutations are relatively rare in CRC, and their prognostic role remains unclear due to limited studies. A previous research in mCRC has associated *NRAS* mutation with shorter OS [47], and other findings indicate that *NRAS* mutation is an independent risk factor for poor OS in stage I-II CRC patients [32]. However, phase III trial-based analyses have found no significant association between *NRAS* mutation and DFS in non-metastatic settings [28,29].

The major limitation of the retrospective analysis is the small sample size. We identified few or no associations between somatic mutations and survival outcomes in this small sample. Yet, we performed a meta-analysis with large sample size to confirm the prognostic impact. Another limitation is the unmatched comparison between the retrospective analysis and meta-analysis according to tumor location and variation in adjuvant therapy. While results of the observational retrospective analysis should be cautiously interpreted, the meta-analysis incorporating *post hoc* analyses of phase III trials provides a higher level of evidence. Our meta-analysis also has several limitations. First, despite being derived from phase III trials, only a proportion, ranging from 26.2% to 98.5%, of patients in each trial were available for post hoc analyses. Second, the anti-EGRF antibody cetuximab was given in two trials [19,20]. Since *KRAS/BRAF* mutations confer resistance to cetuximab, the inclusion of cetuximab-treated patients may lead to an overestimation of the prognostic impact. Third, we did not stratify the analysis according to tumor location or stage. Tumor site (proximal or distal, right-sided or left-sided) and stage (II or III) are independent prognostic indicators of CRC and may be confounders for our survival analysis of *KRAS/BRAF* mutations. For instance, Sinicrope *et al.* observed that *KRAS* mutation had negative impact on OS in distal tumors but not in proximal tumors [19]. Additionally, Domingo *et al*. found that *BRAF* mutation was associated with shorter DFS in stage III but not in stage II tumors [28]. Finally, our results only highlight the prognostic value of *KRAS/BRAF* mutations, without addressing their predictive role in therapy outcomes. For example, among *BRAF* or *PIK3CA*-mutant patients, there was no survival benefit from adding irinotecan to 5-fluorouracil +  leucovorin (5FU/LV) compared to FU/LV alone [17,25].

## Conclusions

In conclusion, our study demonstrates that *KRAS* and *BRAF* mutations are significantly associated with worse OS and DFS in MSS stage II/III CRC receiving adjuvant therapy after curative surgery. Future large-scale, well-designed studies are needed to explore how these findings can inform adjuvant therapy strategies.

## Supporting information

S1 Checklist PRISMA 2020 Checklist. (DOCX)

S1 Data Raw data of the retrospective analysis. (XLSX)

S2 Data Extracted data for meta-analysis. (XLSX)

S1 Table Search strategy in literature databases for meta-analysis. (DOCX)

S2 Table Mutations in the *KRAS*, *NRAS*, *BRAF*, and *PIK3CA* genes in 47 stage II/III CRC patients. (DOCX)

S3 Table Cox regression analysis of OS and DFS in the whole population (n = 47). (DOCX)

S4 Table Cox regression analysis of OS and DFS in MSS population (n = 43). (DOCX)

S5 Table Eligibility assessment for all articles and the exclusion reasons. (XLSX)

S6 Table Quality assessment of included studies using Newcastle-Ottawa Scale for cohort studies. (DOCX)

S1 Fig Forest plot of meta-analysis of the association between *NRAS* mutation and survival.
(PDF)

S2 Fig Forest plot of meta-analysis of the association between *PIK3CA* mutation and disease-free survival.
(PDF)

S3 Fig Forest plot of meta-analysis of the association between *PIK3CA* mutation and overall survival.
(PDF)

S4 Fig Funnel plot of meta-analysis of the association between *KRAS* mutation (A) and *BRAF* mutation (B).
(PDF)
