# Supplementary material for: Prognostic significance of KRAS, NRAS, BRAF, and PIK3CA mutations in stage II/III colorectal cancer: A retrospective study and meta-analysis
Source: PLoS One. 2025 Apr 25;20(4):e0320783. doi: 10.1371/journal.pone.0320783 (PMC12027030; doi:10.1371/journal.pone.0320783)
Supplement: S1 Table — (DOCX) [file pone.0320783.s004.docx]

S1 Table. Search strategy in literature databases for meta-analysis.

| Databases | Search strategy | Items |
| --- | --- | --- |
| PubMed | (‘Colorectal Neoplasms’[Mesh] OR ‘Rectal Neoplasms’[Mesh] OR ‘colorectal cancer’[tiab] OR ‘colon cancer’[tiab] OR ‘colorectal tumor’[tiab] OR ‘colon tumor’[tiab] OR ‘CRC’[tiab]) AND (‘KRAS’[tiab] OR ‘K-RAS’[tiab] OR ‘c-KRAS’[tiab] OR ‘NRAS’[tiab] OR ‘N-RAS’[tiab] OR ‘BRAF’[tiab] OR ‘B-RAF’[tiab] OR ‘PIK3CA’[tiab]) AND (‘stage II’ OR ‘stage III’) | 563 |
| Web of Science | (‘Colorectal Neoplasms’ OR ‘Rectal Neoplasms’ OR ‘colorectal cancer’ OR ‘colon cancer’ OR ‘colorectal tumor’ OR ‘colon tumor’ OR ‘CRC’) AND (‘KRAS’ OR ‘K-RAS’ OR ‘c-KRAS’ OR ‘NRAS’ OR ‘N-RAS’ OR ‘BRAF’ OR ‘B-RAF’ OR ‘PIK3CA’) | 714 |
| Cochrane library | (‘Colorectal Neoplasms’ OR ‘Rectal Neoplasms’ OR ‘colorectal cancer’ OR ‘colon cancer’ OR ‘colorectal tumor’ OR ‘colon tumor’ OR ‘CRC’) AND (‘KRAS’ OR ‘K-RAS’ OR ‘c-KRAS’ OR ‘NRAS’ OR ‘N-RAS’ OR ‘BRAF’ OR ‘B-RAF’ OR ‘PIK3CA’) AND (‘stage II’ OR ‘stage III’) | 186 |
| EMBASE | (‘Colorectal neoplasms’/exp OR ‘Rectal Neoplasms’/exp OR ‘colorectal cancer’ OR ‘colon cancer’ OR ‘colorectal tumor’ OR ‘colon tumor’ OR ‘CRC’) AND (‘KRAS’/exp OR ‘KRAS’ OR ‘K-RAS’ OR ‘c-KRAS’ OR ‘NRAS’/exp OR ‘NRAS’ OR ‘N-RAS’ OR ‘BRAF’/exp OR ‘BRAF’ OR ‘B-RAF’ OR ‘PIK3CA’/exp OR ‘PIK3CA’) AND (‘stage II’ OR ‘stage III’) | 1027 |
