# Supplementary material for: Prognostic significance of KRAS, NRAS, BRAF, and PIK3CA mutations in stage II/III colorectal cancer: A retrospective study and meta-analysis
Source: PLoS One. 2025 Apr 25;20(4):e0320783. doi: 10.1371/journal.pone.0320783 (PMC12027030; doi:10.1371/journal.pone.0320783)
Supplement: S2 Table — (DOCX) [file pone.0320783.s005.docx]

S2 Table. Mutations in *KRAS*, *NRAS*, *BRAF*, and *PIK3CA* genes in 47 stage II/III CRC patients.

| Gene | Mutation | Number (%) |
| --- | --- | --- |
| *KRAS* exon 2 | G12S | 1 (2.1) |
|  | G12C | 2 (4.3) |
|  | G12D | 7 (14.9) |
|  | G12V | 1 (2.1) |
| *KRAS* exon 4 | K117N | 1 (2.1) |
|  | A146T | 1 (2.1) |
|  | A146V | 1 (2.1) |
| *NRAS* exon 2 | G12D | 1 (2.1) |
| *NRAS* exon 3 | Q61H | 1 (2.1) |
| *BRAF* exon 15 | V600E | 1 (2.1) |
| *PIK3CA* exon 1 | R88Q | 1 (2.1) |
| *PIK3CA* exon 9 | E542K | 1 (2.1) |
|  | E545G | 1 (2.1) |
|  | E545K | 1 (2.1) |
| *PIK3CA* exon 20 | H1047L | 1 (2.1) |
|  | H1047R | 2 (4.3) |
