# Supplementary material for: Prognostic significance of KRAS, NRAS, BRAF, and PIK3CA mutations in stage II/III colorectal cancer: A retrospective study and meta-analysis
Source: PLoS One. 2025 Apr 25;20(4):e0320783. doi: 10.1371/journal.pone.0320783 (PMC12027030; doi:10.1371/journal.pone.0320783)
Supplement: S3 Table — (DOCX) [file pone.0320783.s006.docx]

S3 Table. Cox regression analysis of OS and DFS in the whole population (n=47).

| Variables | OS | | | |  | DFS | | | |
| --- | --- | --- | --- | --- | --- | --- | --- | --- | --- |
|  | HR1 (95%CI) | P | HR2 (95%CI) | P |  | HR1 (95%CI) | P | HR2 (95%CI) | P |
| Sex, male vs female | 2.40 (0.27-21.48) | 0.434 | - | - |  | 0.74 (0.25-2.20) | 0.587 | - | - |
| Age, >60 vs ≤60 years | 2.32 (0.26-20.78) | 0.452 | - | - |  | 1.51 (0.45-4.91) | 0.496 | - | - |
| Location, right vs left | 0.60 (0.07-5.38) | 0.649 | - | - |  | 1.02 (0.31-3.33) | 0.973 | - | - |
| Differentiation,  poor vs well to moderate | 0.92 (0.10-8.26) | 0.942 | - | - |  | 0.93 (0.26-3.38) | 0.913 | - | - |
| Stage, III vs II | 2.36 (0.39-14.25) | 0.349 | - | - |  | 3.82 (1.16-12.52) | 0.027 | 3.59 (1.09-11.85) | 0.036 |
| Number of harvested lymph nodes, ≥19 vs <19 | 0.65 (0.11-3.88) | 0.635 | - | - |  | 0.54 (0.18-1.66) | 0.281 |  |  |
| *KRAS*, mutant vs wildtype | 1.33 (0.22-7.98) | 0.755 | - | - |  | 0.92 (0.28-2.98) | 0.884 | - | - |
| *NRAS*, mutant vs wildtype | NA | 0.611^#^ | - | - |  | NA | 0.405^#^ | - | - |
| *BRAF*, mutant vs wildtype | NA | 0.534^#^ | - | - |  | 0.94 (0.12-7.38) | 0.956 | - | - |
| *PIK3CA*, mutant vs wildtype | 4.27 (0.71-25.61) | 0.113 | - | - |  | 2.39 (0.63-9.02) | 0.198 | - | - |
| Concurrent mutations, yes vs no | 5.61 (0.93-33.74) | 0.059 | - | - |  | 3.29 (0.87-12.43) | 0.079 | 2.84 (0.75-10.79) | 0.126 |
| MSI-H vs MSS | NA | 0.515^#^ | - | - |  | NA | 0.291^#^ | - | - |

^#^ Log-rank test p value.

CI: confidence interval; DFS: disease-free survival; HR1: hazard ratio of univariate analysis; HR2: hazard ratio of multivariate analysis; MSI-H: microsatellite instability-high; MSS: microsatellite stability; NA: Cox model not applicable due to lack of events; OS: overall survival.
