# Supplementary material for: Prognostic significance of KRAS, NRAS, BRAF, and PIK3CA mutations in stage II/III colorectal cancer: A retrospective study and meta-analysis
Source: PLoS One. 2025 Apr 25;20(4):e0320783. doi: 10.1371/journal.pone.0320783 (PMC12027030; doi:10.1371/journal.pone.0320783)
Supplement: S4 Table — (DOCX) [file pone.0320783.s007.docx]

S4 Table. Cox regression analysis of OS and DFS in MSS population (n=43).

| Variables | OS | | | |  | DFS | | | |
| --- | --- | --- | --- | --- | --- | --- | --- | --- | --- |
|  | HR1 (95%CI) | P | HR2 (95%CI) | P |  | HR1 (95%CI) | P | HR2 (95%CI) | P |
| Sex, male vs female | 2.45 (0.27-21.96) | 0.422 | - | - |  | 0.77 (0.26-2.29) | 0.640 | - | - |
| Age, >60 vs ≤60 years | 2.65 (0.30-23.71) | 0.384 | - | - |  | 1.73 (0.53-5.65) | 0.361 | - | - |
| Location, right vs left | 0.69 (0.08-6.20) | 0.743 | - | - |  | 1.17 (0.36-3.83) | 0.789 | - | - |
| Differentiation,  poor vs well to moderate | 1.41 (0.16-12.65) | 0.761 | - | - |  | 1.31 (0.36-4.76) | 0.683 | - | - |
| Stage, III vs II | 2.09 (0.35-12.63) | 0.420 | - | - |  | 3.42 (1.04-11.23) | 0.042 | 3.01 (0.90-10.11) | 0.074 |
| Number of harvested lymph nodes, ≥19 vs <19 | 0.77 (0.13-4.59) | 0.771 | - | - |  | 0.63 (0.21-1.94) | 0.424 | - | - |
| *KRAS*, mutant vs wildtype | 1.32 (0.22-7.92) | 0.763 | - | - |  | 0.90 (0.28-2.92) | 0.857 | - | - |
| *NRAS*, mutant vs wildtype | NA | 0.595^#^ | - | - |  | NA | 0.385^#^ | - | - |
| *BRAF*, mutant vs wildtype | NA | 0.516^#^ | - | - |  | 0.86 (0.11-6.69) | 0.882 | - | - |
| *PIK3CA*, mutant vs wildtype | 5.12 (0.85-30.79) | 0.074 | - | - |  | 2.98 (0.79-11.27) | 0.107 | - | - |
| Concurrent mutations, yes vs no | 6.91 (1.15-41.73) | 0.035 | - | - |  | 4.09 (1.08-15.46) | 0.038 | 3.14 (0.81-12.14) | 0.097 |

^#^ Log-rank test p value.

CI: confidence interval; DFS: disease-free survival; HR1: hazard ratio of univariate analysis; HR2: hazard ratio of multivariate analysis; MSS: microsatellite stability; NA: Cox model not applicable due to lack of events; OS: overall survival.
