# Supplementary material for: Prognostic significance of KRAS, NRAS, BRAF, and PIK3CA mutations in stage II/III colorectal cancer: A retrospective study and meta-analysis
Source: PLoS One. 2025 Apr 25;20(4):e0320783. doi: 10.1371/journal.pone.0320783 (PMC12027030; doi:10.1371/journal.pone.0320783)
Supplement: S6 Table — (DOCX) [file pone.0320783.s009.docx]

S6 Table. Quality assessment of included studies using Newcastle-Ottawa Scale for cohort studies.

| Study | Selection domain | | | | Comparability domain | Outcome domain | | | Total stars |
| --- | --- | --- | --- | --- | --- | --- | --- | --- | --- |
|  | Item 1 | Item 2 | Item 3 | Item 4 | Item 5 | Item 6 | Item 7 | Item 8 |  |
| French, 2008 | * | * | * | * |  | * | * | * | 7 |
| Hutchins, 2011 | * | * | * | * |  | * | * | * | 7 |
| Ogino, 2012 | * | * | * | * | ** | * | * | * | 9 |
| Gavin, 2012 | * | * | * | * |  | * | * | * | 7 |
| Roth, 2012 | * | * | * | * | ** | * | * | * | 9 |
| Ogino, 2013 | * | * | * | * | ** | * | * | * | 9 |
| Pentheroudakis, 2015 | * | * | * | * |  | * | * | * | 7 |
| Sinicrope, 2015 | * | * | * | * | ** | * | * | * | 9 |
| Andre, 2015 | * | * | * | * |  | * | * | * | 7 |
| Taieb, 2016 | * | * | * | * | ** | * | * | * | 9 |
| Domingo, 2018 | * | * | * | * | ** | * | * | * | 9 |
| Shida, 2023 | * | * | * | * | * | * | * | * | 8 |
| Nowak, 2024 | * | * | * | * | ** | * | * | * | 9 |

Item 1: Representativeness of the exposed cohort.

Item 2: Selection of the non-exposed cohort.

Item 3: Ascertainment of exposure.

Item 4: Demonstration that outcome of interest was not present at start of study.

Item 5: Comparability of cohorts on the basis of the design or analysis. Adjustment for clinicopathological features in multivariate analysis (1 star); Adjustment for MSI status in multivariate analysis (1 star).

Item 6: Assessment of outcome.

Item 7: Was follow-up long enough for outcome to occur?

Item 8: Adequacy of follow-up of cohorts.
