# Supplementary figures and images for: Prognostic significance of KRAS, NRAS, BRAF, and PIK3CA mutations in stage II/III colorectal cancer: A retrospective study and meta-analysis
Source: PLoS One. 2025 Apr 25;20(4):e0320783. doi: 10.1371/journal.pone.0320783 (PMC12027030; doi:10.1371/journal.pone.0320783)

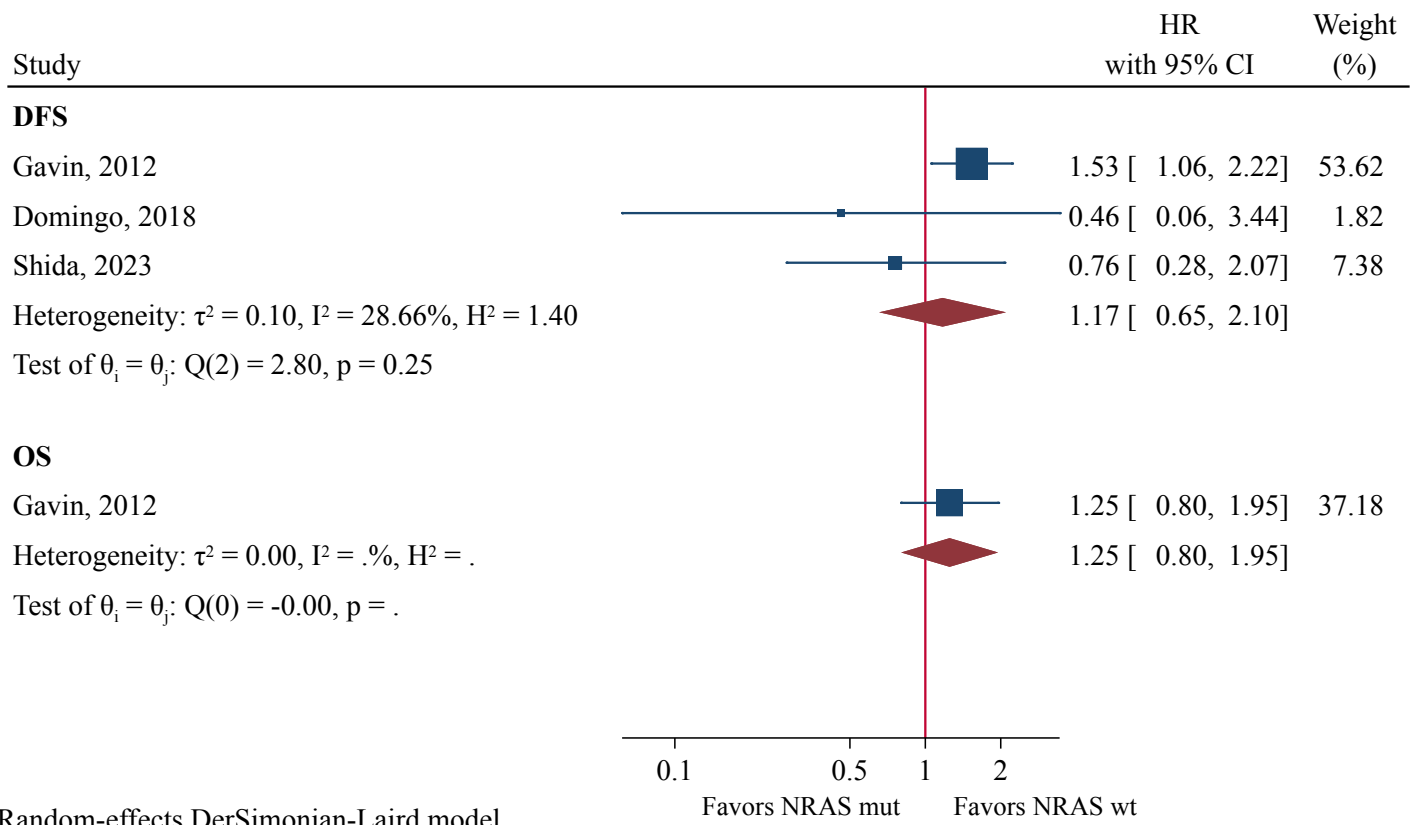

Supplement: S1 Fig — Forest plot of meta-analysis of the association between NRAS mutation and survival. (PDF) [file pone.0320783.s010.pdf]

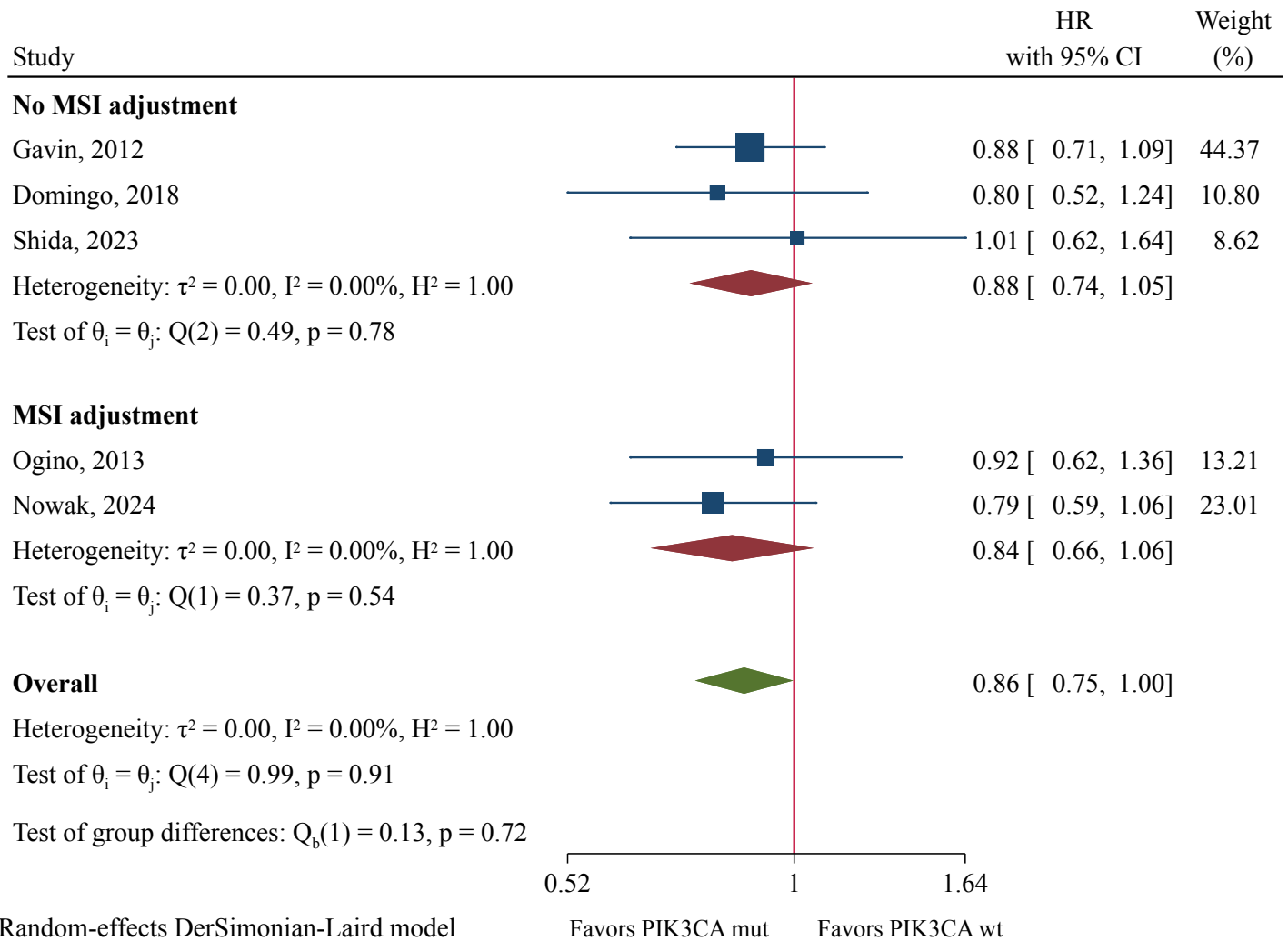

Supplement: S2 Fig — Forest plot of meta-analysis of the association between PIK3CA mutation and disease-free survival. (PDF) [file pone.0320783.s011.pdf]

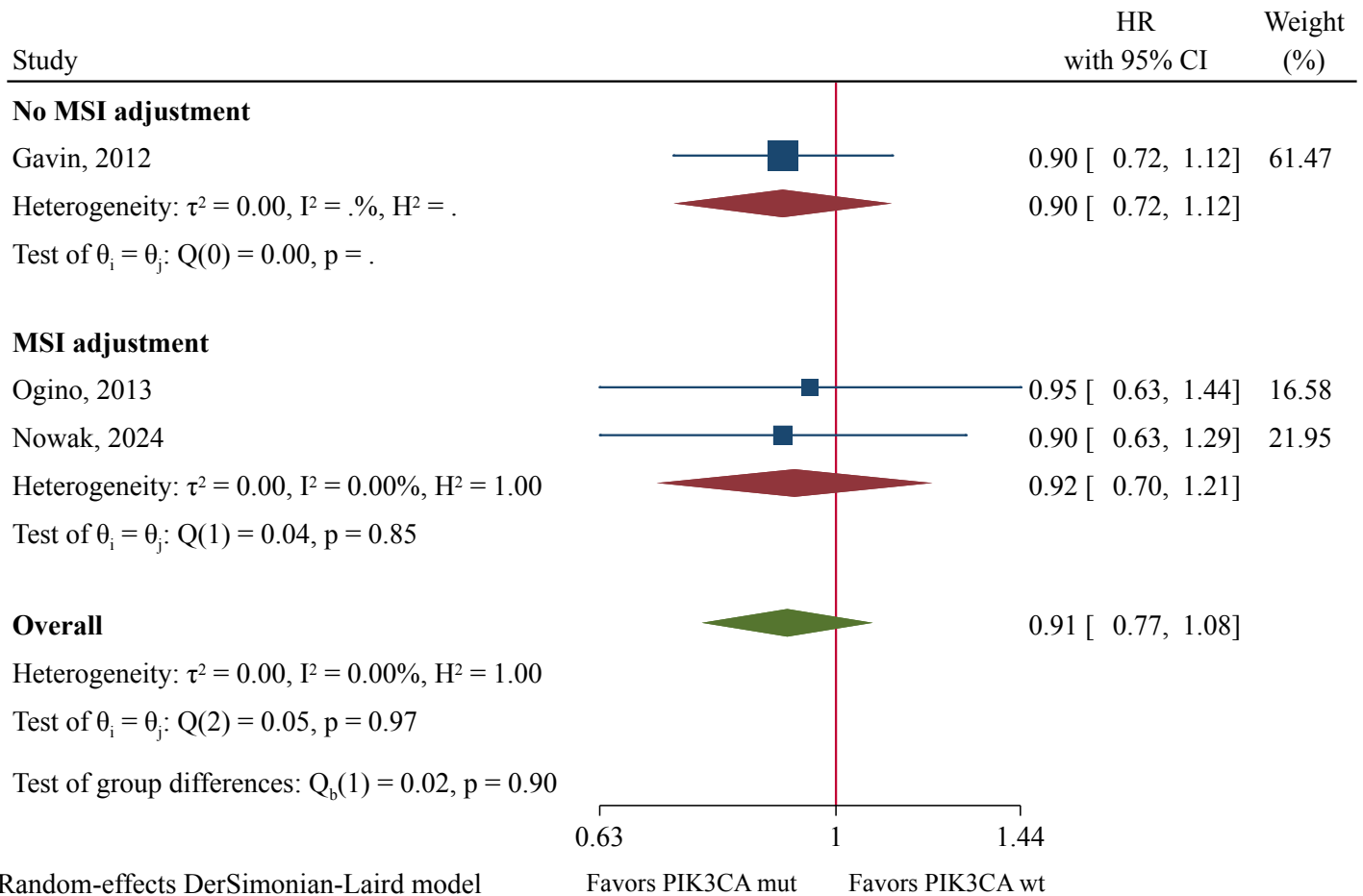

Supplement: S3 Fig — Forest plot of meta-analysis of the association between PIK3CA mutation and overall survival. (PDF) [file pone.0320783.s012.pdf]

A

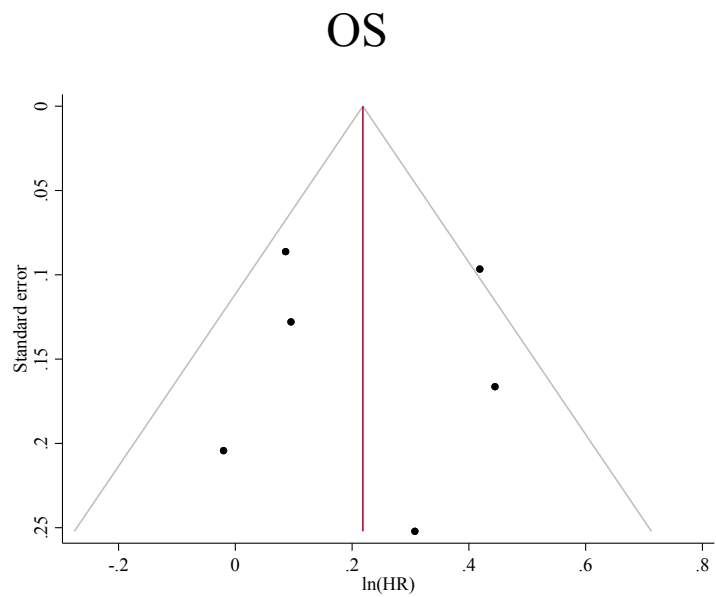

DFS

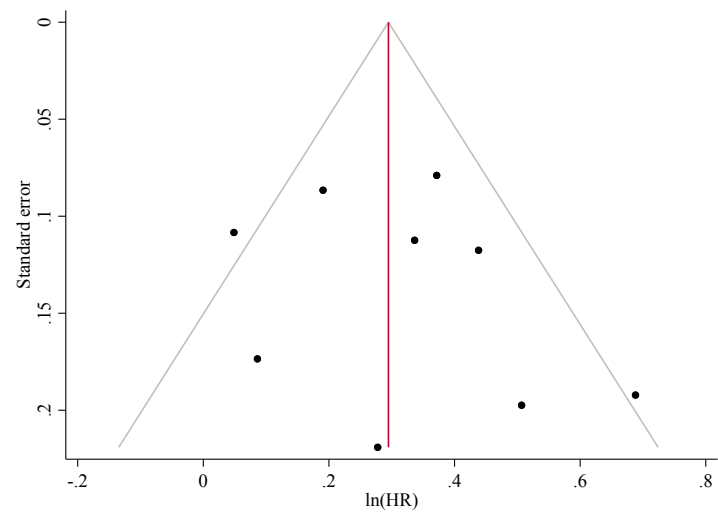

B

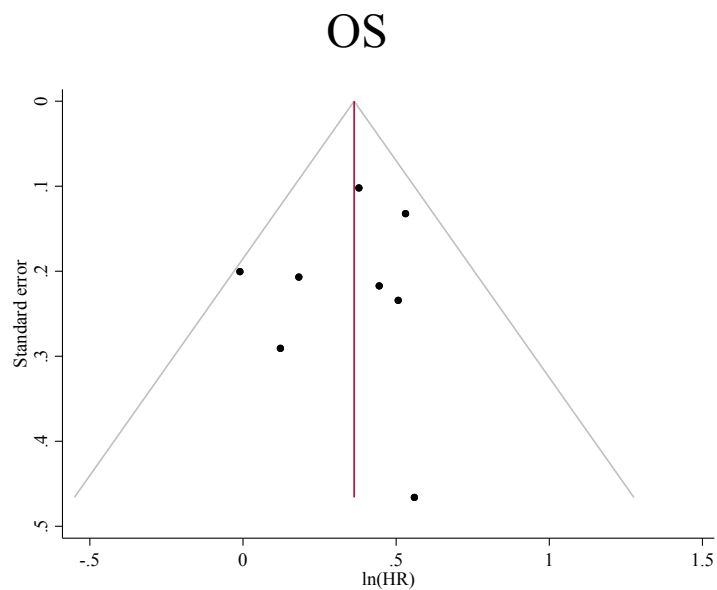

DFS

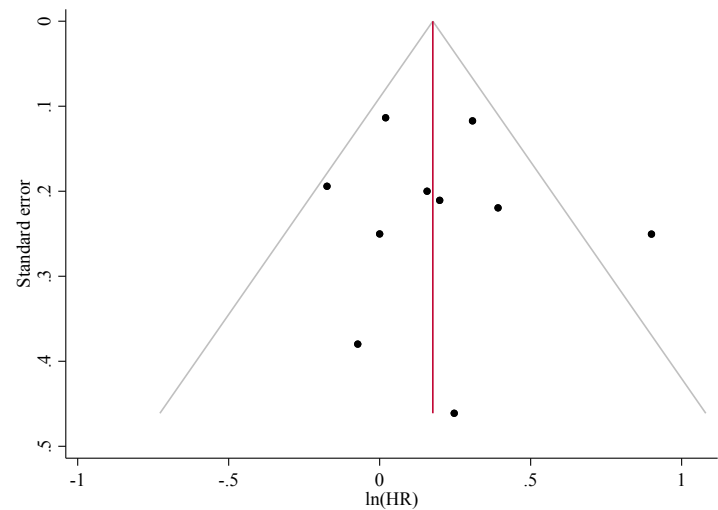

Supplement: S4 Fig — Funnel plot of meta-analysis of the association between KRAS mutation (A) and BRAF mutation (B). (PDF) [file pone.0320783.s013.pdf]
